# DNA Methylation of *PTGIS* Enhances Hepatic Stellate Cells Activation and Liver Fibrogenesis

**DOI:** 10.3389/fphar.2018.00553

**Published:** 2018-05-28

**Authors:** Xue-yin Pan, Yang Yang, Hong-wu Meng, Hai-di Li, Xin Chen, Hui-min Huang, Fang-tian Bu, Hai-xia Yu, Qin Wang, Cheng Huang, Xiao-ming Meng, Jun Li

**Affiliations:** ^1^The Key Laboratory of Major Autoimmune Diseases, Anhui Province, Anhui Institute of Innovative Drugs, School of Pharmacy, Anhui Medical University, Hefei, China; ^2^The Key Laboratory of Anti-inflammatory of Immune Medicine, Ministry of Education, Hefei, China; ^3^Institute for Liver Diseases of Anhui Medical University, Hefei, China

**Keywords:** DNA methylation, gene expression, proliferation, apoptosis, hepatic stellate cells, PTGIS

## Abstract

The activation of hepatic stellate cells (HSCs) is a central event in the progression of liver fibrosis. Multiple studies proved that DNA methylation might accelerate HSCs activation. However, the specific pathogenesis of liver fibrosis remains not fully addressed. Our laboratory performed Genome methylation screening to find out the methylated gene in mice with liver fibrosis. The pilot experiments showed that the promoter of *prostacyclin synthase* (*PTGIS*) gene was hypermethylated in CCl_4_-induced liver fibrosis mouse model. Moreover, the down-regulated PTGIS expression can be restored by DNMTs-RNAi and 5-aza-2-deoxycytidine (5-azadC), an inhibitor of DNA methyltransferase (DNMTs). Methylation-specific PCR (MSP) showed that the methylation status of PTGIS in HSC-T6 cells cultures with TGF-β1 (10 ng/mL) was elevated compared with control group. Chromatin immunoprecipitation (ChIP) assay indicated that PTGIS methylation was mainly induced by DNMT1 and DNMT3b. We further investigated the function of PTGIS in liver fibrosis by Recombinant Hepatic-adeno-associated virus (rAAV8)-PTGIS overexpression. The data indicated that overexpression of PTGIS in mouse liver accompanied by elevated apoptosis-related proteins expression in primary HSCs. Conversely, PTGIS silencing mediated by RNAi enhanced the expression of α-SMA and COL1a1 *in vitro*. Those results illustrated that adding PTGIS expression inhibits the activation of HSCs and alleviates liver fibrosis. Therefore, our study unveils the role of PTGIS in HSCs activation, which may provide a possible explanation for CCl_4_-mediated liver fibrosis.

## Introduction

Liver fibrosis, characterized with the progressively increased accumulation of ECM compounds in liver, is a wound-healing response to various chronic hepatic injuries including virus infection, alcohol abuse and high-fat diet (Bataller and Brenner, 2005; Hernandez-Gea and Friedman, 2011; Trautwein et al., 2015). Without effective therapeutic methods, liver fibrosis can eventually develop into liver cirrhosis and hepatic cancer(Bataller and Brenner, 2005; Trautwein et al., 2015), often requiring liver transplant, pose a huge heath burden on the global community. HSCs, which located in the perisinusoidal space of Disse, characterized by the presence of cytoplasmic perinuclear droplets that are laden with retinyl (vitamin A) esters (Senoo et al., 1998; Moreira, 2007; Friedman, 2008a). After liver injury, the quiescent HSCs (qHSCs) are trans-differentiate into alpha smooth muscle actin (α-SMA)-positive myofibroblast-like cells (Rippe and Brenner, 2004; Mallat and Lotersztajn, 2013). The hepatic architecture will be disturbed by the prolonged and repeated accumulation of ECM proteins by which can form fibrotic scars and nodules, leading to hepatic dysfunction ultimately (Hernandez-Gea and Friedman, 2011; Trautwein et al., 2015). TGF-β1, an inflammatory cytokines is a major pro-fibrogenic cytokine, can up-regulate α-SMA and type I collagen synthesis by HSCs-derived myofibroblast (Hellerbrand et al., 1999). The mechanisms governing the role of the HSCs in fibrosis are still not fully defined.

Increasing evidence suggests that epigenetic modifications play an important role in liver fibrosis. DNA methylation and histone modifications are two key players in epigenetic regulation of gene expression in mammalian cells (Subramaniam et al., 2014). DNA methylation often catalyzed by DNMTs family including DNMT1, DNMT3a and DNMT3b (Anestopoulos et al., 2015). Abnormal patterns of DNA methylation occuring in various liver diseases have been recognized over the last years, such as liver inflammation, fibrosis and cancer (Bian et al., 2014; Wu et al., 2017). Accumulating evidence have demonstrated that DNA methylation in hepatic fibrosis facilitate activation of HSCs and promote the progression of liver fibrogenesis (Bian et al., 2014; Cai et al., 2016; Wu et al., 2017). Therefore, genome-wide analysis of DNA methylation statues in fibrotic organs (e.g., liver, kidney, heart, lung and intestines) will favor to identify the novel DNA methylation biomarkers for fibrotic diseases of different organs. In this paper, we argue that DNA methylation is activated in HSCs in the presence of specific stimuli that correspond to liver fibrosis response.

*Prostacyclin synthase (PTGIS, PGIS, CYP8A1)* is a member of family 8 (CYP8) in the cytochrome P450 superfamily. PTGIS catalyzes the conversion of PGH_2_ to prostacyclin (PGI_2_). And it is a potent mediator of vasodilation and inhibitor of platelet aggregation and cell proliferation (Yokoyama et al., 1996; Tuder et al., 1999; Cho et al., 2015). Various studies have highlighted the importance of the PTGIS expression in preventing tumor growth and progression (Keith et al., 2002, 2004; Pradono et al., 2002). For example, Keith et al. (2002) demonstrated that over-expression of PTGIS can prevent the development of murine lung tumors and inhibit lung carcinogenesis in tobacco-smoke exposed mice (Keith et al., 2004). Moreover, Keith et al. found that PGI_2_, the catalysate of PTGIS, appears to exert anti-proliferative effects (Paz et al., 2003). Moreover, Frigola et al. (2005) reported that *PTGIS* promotor hypermethylation is a frequent event in colorectal cancer and PTGIS expression was restored when cultured colorectal cancer cell line HCT116 with the demethylating agent 5-azadC (Sadler et al., 2016). In short, those studies suggested that PTGIS exerts an important role in many physiology and pathology process and its expression levels was influenced by DNA methylation. Nevertheless, it is not clear whether changes in PTGIS expression is involved in liver fibrosis.

In the previous study of our laboratory, the genomic methylation analysis results shown that *PTGIS* gene was methylated in mice with liver fibrosis. In this study, we sought to define the potential roles of PTGIS in regulating HSCs activation and survival and the molecular mechanism underlying this regulation in liver fibrosis.

## Materials and Methods

### Animals, Mouse Models of Liver Fibrosis

Normal male C57BL/6J (18–22 g) mice were obtained from the Experimental Animal Center of Anhui Medical University used for CCl_4_-induced liver fibrosis model. The animal experimental procedures were reviewed and approved by the University Animal Care and Use Committee. Mice were randomly divided into two groups (eight mice per group) including vehicle group and model group. Hepatic fibrosis was generated by biweekly intraperitoneal injection of carbon tetrachloride (CCl_4_, 10% in olive oil) at a dose of 0.01 mL/g/mouse for 6 weeks. Mice in vehicle group were treated intraperitoneal injection with the same volume of olive oil at the same time intervals. Six weeks later, mice were killed under anesthesia. Samples of blood and liver tissues were collected for further analysis.

### Recombinant-Adeno-Associated-Virus-Mediated PTGIS Overexpression in Mice

Mouse PTGIS overexpression plasmid labeled with green fluorescent protein (GFP) was obtained from Genechem Co., Ltd. (Shanghai, China). PTGIS plasmid was packaged with Recombinant-adeno-associated-virus 8 for overexpression PTGIS *in vivo*. C57BL/6 mice (18–22 g) were housed at the Animal Experimental Center for 1 week in order to adapt to the environment. Then the tails of mice were wiped with alcohol to expand the tail vein for injection. Mice were slowly injected with 100 μL Recombinant-adeno-associated-virus-packaged PTGIS overexpression plasmid with a concentration of 1 × 10^11^ v.g/mL/mouse through tail vein using 0.5 mL insulin syringe. One week later, mice were intraperitoneally injected with either 10% CCl_4_ or the equal volume of olive oil for further analysis.

### Serum Level of ALT/AST Activity Assay

Serum ALT and AST activities were detected by alanine aminotransferase/aminotransferase Assay Kits (Nanjing Jiancheng Bioengineering Institute) according to the manufacturer’s instructions. The absorbance at 510 nm was obtained with a Multiskan MK3 (Biotek, United States).

### Primary HSCs Isolation

Mice were chosen randomly from the vehicle group and CCl_4_-treated group (*n* = 8/group). The primary HSCs were perfused from liver tissue of mice in the above two groups. Briefly, a 20-G catheter was put through mouse the portal vein after the mice were anesthetized, then the inferior vena cava were cut. The liver was perfused with PB. Subsequently, the liver was perfused with digestion buffer. After digestion, the liver was disrupted in 1% BSA solution. Single cells were passed through 200-mesh sieve cell strainer. Cells suspension was centrifuged at 4°C, 50 *g*, 2 min. The supernate were collected to a new 15 mL centrifuge tube, and centrifuged at 4°C, 760 *g*, 10 min. Discarding the supernate and resuspending the sediment with 4 mL DMEM. The cells suspension was then centrifuged at 4°C, 760 *g*, 7 min. Discarding the supernate and resuspending the sediment with 2 mL DMEM. Subsequently, cells suspension and Nycodenz (Sigma, GER) were mixed at a density of 1.040–1.060 g/mL. To create discontinuous gradient, the cell-Nycodenz mixture was covered with 1mL Hank’s fluid (Gibco, United States). Then centrifuged at 20°C, 1350 *g*, 18 min. After centrifugation, the primary HSCs in the interface were collected to a new 15 mL centrifuge tube for following analysis. Then we can get the cells pellet after the cells suspension were centrifuged at 20°C, 1350 g, 5 min. Discarding the supernate and resuspending the cells pellet with 3 mL DMEM. Then the cells suspension can be cultured in the culture bottles. The preparation methods of solution used in primary HSCs isolation were listed in **Table 1**.

**Table 1:** The preparation methods of solution used in primary HSCs isolation

(1) PB solution	
**Compounds**	**Volume**
PBC solution	40 mL
ddH_2_O	Up to 1 L

**Compounds**	**Volume/Quality**
(2) PBC solution	
NaCl	103.75 g
KCl	6.25 g
Hepes	28.7 g
1M NaOH solution	75 mL
ddH_2_O	Up to 500 mL
The solution preparation method of PBC: dissolve NaCl (103.75 g), KCl (6.25 g) and Hepes (28.70 g) in H2O (350 mL) while stirring. When all has been dissolvedadd 1M NaOH (75 mL). Add H2O to a total volume of 500 mL.	
(3) 1% BSA solution	
BSA	1 g
PB solution	Up to 100 mL
(4) Digestion buffer	
type IV collagenase	35 mg
Pronase	35 mg
1M CaCl_2_ solution	1 mL
PBC solution	99 mL
(5) Nycodenz solution	
Nycodenz	28.7 g
GBSS solution	Up to 100 mL
(6) GBSS solution	
KCl	0.37 g
CaCl_2_	0.225 g
MgCl_2_.6H_2_O	0.21 g
MgSO_4_	0.0342 g
KH_2_PO_4_	0.03 g
NaHCO_3_	2.27 g
NaH_2_PO_4_	0.1196 g
Glucose	1.0 g
ddH_2_O	Up to 1000 mL

### Histopathology

The middle portion of the left lobe of the liver of each C57BL/6J mice was excised and sectioned and then perfused in 4% paraformaldehyde for at least 48 h. After fixation, the tissues were embedded in paraffin, and 5 μm thick sections were then stained with H&E and Masson’s trichrome for morphological analysis and locating collagen expression by using the standard protocols, respectively.

### Immunofluorescence Staining

For immunofluorescence staining, sections were blocked with 10% BSA blocking solution in order to avoid unspecific staining. Then, the sections were incubated with rabbit polyclonal primary antibodies for PTGIS (1:50) mouse monoclonal primary antibodies for α-SMA (1:50). Sections were incubated with both primary antibodies over-night at 4°C, followed by a mixture of anti-rabbit FITC (green, 1:200) and anti-mouse Cy-3 (red, 1:200) conjugated secondary antibodies for 2 h at room temperature. α-SMA and PTGIS expression was visualized by 3, 3′-diaminobenzidine tetrahydrochloride (DAB) staining. Then the stained sections were examined with Inverted fluorescence Microscope (OLYMPUS IX83, Tokyo, Japan).

### Cell Culture

The HSC-T6 cells line was obtained from the Type Culture Collection of the Chinese Academy of Sciences (Shanghai, China). HSC-T6 cells (Fumeng Gene, China), an immortalized rat HSCs, were cultured in DMEM (Keygen Bio, China) supplemented with 5%(v/v) Fetal Bovine Serum (FBS, Biological Industries, Israel). HSC-T6 cells were incubated at 37°C with 5% CO_2_ and propagated every 2 days. HSC-T6 cells were activated by 10 ng/mL TGF-β1 (Peprotech, United States) (Cai et al., 2016).

### Total RNA Isolation and Quantitative Real-Time PCR

After splitting by TRIzol reagents (Invitrogen, United States), total RNA was extracted from mice primary HSCs (*n* = 8/group) and HSC-T6 cells. RNA quantification was determined by Nanodrop 2000 (Thermo Scientific, United States). The mRNA levels of α-SMA, PTGIS, COL1a1, β-actin and GAPDH were determined by RT-qPCR. The primer sequences (Sangon Biotech, China) were listed in **Table 2**. The mRNA level of α-SMA, PTGIS and COL1a1 were normalized by β-actin (rat) or GAPDH (mice). All samples were performed in triplicate and repeated at least three times.

**Table 2:** Primers used in RT-qPCR.

Gene	Forward Primer (5′ → 3′)	Reverse Primer (5′ → 3′)
Mouse		
PTGIS	TCCTCAAGAATCCGGAAGCC	TCTTCTGTGGGAGTGTGGTC
Colla1	TGTAAACTCCCTCCACCCCA	TCGTCTGTTTCCAGGGTTGG
α-SMA	CGGGCTTTGCTGGTGATG	CCCTCGATGGATGGGAAA
GAPDH	GGACCTCATGGCCTACATGG	TAGGGCC TCTCTTGCTCAGT
Rat		
PTGIS	TTATTACTGTTGCTGCTGCT	ATCCTGGTAAGGAAGCTGGC
Colla1	GATCCTGCCGATGTCGCTAT	TGTAGGCTACGCTGTTCTTGCA
α-SMA	CGAAGCGCAGAGCAAGAGA	CATGTCGTCCCAGTTGGTGAT
β-actin	CCCATCTATGAGGGTTACGC	TTTAATGTCACGCACGATTTC

### Transfection With PTGIS Plasmid

Adding expression of PTGIS in HSC-T6 cells was mediated by rat-derived PTGIS overexpression plasmid. The transfection efficiency was measured by Western blot and RT-qPCR analysis. Plasmid constructs were purchased from GenePharma Co., Ltd. The transfection procedure were performed as follows. Briefly, 3 × 10^5^/mL HSC-cells were seeded in 6-well plates, cultured in DMEM contain 5% FBS for 12 h as described in Section “Cell Culture.” After adhere to the wall, HSC-T6 cells were transfected with 1000 ng/mL pEX-2-Control or pEX-2-PTGIS overexpression plasmid mixed with Lipo2000 transfection reagent (Invitrogen, Carlsbad, CA, United States) according to the manufacturer’s instruction. Cells were incubated with Opti-MEM at 37°C and 5% CO_2_ for 6 h. HSC-T6 cells transfected with PTGIS-plasmid were then cultured in DMEM containing 5% FBS and treated with TGF-β1 (10 ng/mL) for 24 h and consequently harvested for analysis with Western blotting, RT-qPCR and other experiments. All experiments were repeated at least three times.

### RNA Interference Analysis

Transfection of HSC-T6 cells was conducted with lipofection technique, using Lipofectamine TM2000. Small interfering RNA (siRNA) oligonucleotides used for knockdown gene and a negative scrambled siRNA was used in parallel were synthesized by GenePharma (Shanghai, China). The siRNA sequences used in RNA interference analysis were listed in **Table 3**. All siRNA were purchased from GenePharma Co., Ltd. Briefly, 3 × 10^5^/mL HSC-T6 cells were seeded in six-well plates, cultured with DMEM contain 5% FBS for 12 h. Subsequently, HSC-T6 cells were transfected with 1000 ng/mL PTGIS-RNAi or Scrambled-RNAi and mixed with lipo2000 transfection reagent (Invitrogen, Carlsbad, CA, United States) according to the manufacturer’s instruction. Cells were incubated with Opti-MEM at 37°C and 5% CO_2_ for 6 h. HSC-T6 cells transfected with PTGIS-RNAi or Scrambled-RNAi were then cultured in DMEM containing 5% FBS and treated with TGF-β1 (10 ng/mL) for 24 h and consequently harvested for analysis with western blot and RT-qPCR and other experiments. The transfectiong efficiency were determined by Western blot and RT-qPCR analysis. All experiments were performed in triplicate and repeated at least three times.

**Table 3:** The siRNA sequences used in RNA interference analysis.

Gene	Sense (5′ → 3′)	Antisense (5′ → 3′)
RAT		
PTGIS	GCUCACGGAAGCCAUGUAUTT	AUACAUGGCUUCCGUGAGCTT
Negative control	UUCUCCGAACGUGUCACGUTT	ACGUGACACGUUCGGAGAATT
DNMT1	CCCAGAGUAUGCACCAAUATT	UAUUGGUGCAUACUCUGGGTT
DNMT3a	GCGUCACACAGAAGCAUAUTT	AUAUGCUUCUGUGUGACGCTT
DNMT3b	AGAUGACAGGUGCCCAGAGUU	CUCUGGGCACCUGUCAUGUUU
Negative control	UUCUCCGAACGUGUCACGUTT	ACGUGACACGUUCGGAGAATT

### CCK-8 Analysis

The proliferation of HSC-T6 cells was determined by Cell Counting Kit-8 (CCK-8) analysis. Hundred microliter HSC-T6 cells suspension were seeded in 96-well culture plates at a density of 5 × 10^3^ per well, and the edge wells were filled with sterile PBS. After attachment, HSC-T6 cells were transfected with pEX-2-PTGIS or pEX-2-Control or PTGIS-RNAi or Scrambled-RNAi for 6 h in Opti-MEM. Then cells were cultured in DMEM containing 5% FBS. The transfected HSC-T6 cells were then treated with TGF-β1 (10 ng/mL) for 24 h. Then, 10 μL CCK-8 (Sigma, United States) was added for 4 h. The value of absorbance (A) was examined at the wavelength of 490 nm. Cell viability = the A value of transfection wells/the A value of control wells ^∗^ 100%. All experiments were performed in triplicate and repeated at least three times.

### Flow Cytometer Analysis

Cell cycle analysis: HSC-T6 cells were trypsinized and collected in 15 mL centrifuge tubes. The cells suspension was centrifuged (1000 *g*, 3–5 min), mixed sufficiently and fixed in cold ethanol (70%, 1 mL) at 4°C for 12–24 h. After being centrifuged and resuspended, the HSC-T6 cells were added with 0.5 mL mixture (RNase and PI) and incubated for 30 min at 37°C in dark place. Flow cytometer (BD Biosciences) was used to detect the red fluorescence and scattered light at 488 nm wavelength. The analysis of DNA content and scattering light was processed by using the ModFit software (Verity Software House, United States). All experiments were repeated for three times. Cell apoptosis analysis: HSC-T6 cells were collected from suspension by centrifugation, and binding buffer (400 μL) was added to resuspend the cells (about 1^∗^10^6^/mL). Then, 5 μL Annexin V-FITC (5 min, 2–8°C, dark) and PI were added (5 min, 2–8°C, dark), successively. When the preparation was completed, cells suspension were detected by flow cytometer (BD Biosciences) within 1 h and data fitting was made by FlowJo software (TreeStar, United States). All samples were assayed in triplicates.

### Western Blot Analysis

Total protein from cultured HSC-T6 cells and primary HSCs were extracted with RIPA lysis buffer (contained 1% PMSF) (Beyotime, China). Protein of each sample 30–50 μg was separated by SDS-PAGE gel (10%) and then transferred onto PVDF membranes (Millipore Corp, Billerica, MA, United States). The transferred membranes were blocked in 5% skim milk for 3 h in order to break non-specific binding. Subsequently, membranes were incubated with the primary antibody against PTGIS, COL1a1, α-SMA, β-actin, C-myc, Cyclin D1, Bax, Bcl-2, cleaved-casepase 3, DNMT1, DNMT3a and DNMT3b overnight at 4°C, followed by incubation with secondary antibody at room temperature for 1 h. Signals were captured with Bioshine ChemiQ image system. The intensities of each western blotting band were quantified and analyzed by using the Image J software (NIH, Bethesda, MD, United States). The characteristics of antibodies were listed in **Table 4**.

**Table 4:** The characteristics of antibodies.

Protein	Application	Origin	Dilution
PTGIS	WB &IHC&IF	sc-20933, Santa Cruz, CA, United States	1:200 &1:50&1:50
COL1a1	WB	bs10423R, Bioss, China	1:300
α-SMA	WB &IHC&IF	bs0189R, Bioss, China	1:300 &1:200&1:50
DNMT1	WB	ab13537, Abcam, United Kingdom	1:800
DNMT3a	WB	ab13888, Abcam, United Kingdom	1:800
DNMT3b	WB	ab2851, Abcam, United Kingdom	1:800
C-myc	WB	D3N8F, Cell Signaling Technology, United States	1:800
CyclinD1	WB	92G2, Cell Signaling Technology, United States	1:800
Bax	WB	2772s, Cell Signaling Technology, United States	1:800
Bcl-2	WB	ab194583, Cell Signaling Technology, United States	1:800
Casepase3	WB	9662, Cell Signaling Technology, United States	1:800

### Immunohistochemistry

Briefly, Liver tissues were firstly fixed in 10% neutral buffered formalin solution, follow embedded in paraffin and stained for routine histology. Slides were dewaxed in xylene and dehydrated in gradient alcohol, and antigen retrieval was obtained by microwaving in citric buffer for 15 min. After antigen retrieval, sections were deparaffinized and treated with 0.3% hydrogen peroxide for 15 min. Then, the sections were further blocked by 5% BSA and incubated with primary antibody against PTGIS (1:50) and α-SMA (1:200) overnight at 4°C. Follow on, the sections were incubated with biotinylated secondary antibody for 60 min at room temperature after rinsing. PTGIS and α-SMA expression was visualized by using 3,3-diaminobenzidine tetrahydrochloride (DAB) staining. Finally, the sections were mounted by gums and subjected to microscopic examination.

### Statistical Analysis

Statistical Analysis Data are expressed as the mean ± SEM. One-way analysis of variance followed by the Newman–Keuls *post hoc* test (Prism 5.0 GraphPad Software, Inc., San Diego, CA, United States) was used to analyze results. All other protocols, which include MSP assay, ChIP assays are detailed in the Supplementary Materials and Methods.

## Results

### Expression of PTGIS Was Down-Regulated in CCl_4_-Induced Fibrotic Model

**FIGURE 1:**
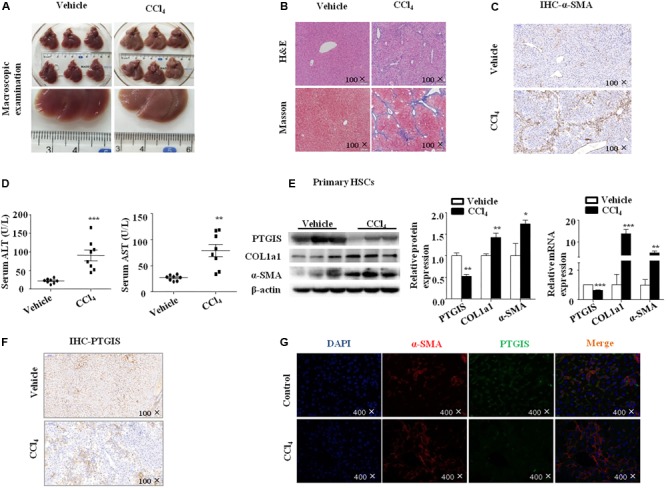
The expression of PTGIS in fibrotic model in C57BL/6J mice. **(A)** Macroscopic examination of fresh liver tissue without fixation from C57BL/6J mice in Vehicle group and Model group. **(B)** Pathology observation of the mouse liver sections stained with H&E staining, Masson staining (100×). **(C)** The expression of α-SMA were determined by immunohistochemistry (IHC) staining (100×). **(D)** Serum ALT and AST levels of mice between vehicle group and model group were determined, ^∗∗^*p* < 0.01, ^∗∗∗^*p* < 0.001 vs. Vehicle group, *n* = 8/group. **(E)** The protein and mRNA levels of PTGIS, COL1a1 and α-SMA in primary HSCs isolated from fibrotic livers were analyzed by western blot and RT-qPCR analysis, representative blots of three of three independent experiments are shown, ^∗^*p* < 0.05, ^∗∗^*p* < 0.01 vs. Vehicle group. **(F)** The expression levels of PTGIS in fibrotic liver were determined by Immunohistochemistry staining (100×). **(G)** Double immunofluorescence staining of α-SMA (red) and PTGIS (green), representative views from Vehicle group and CCl_4-_treated mice were presented (400×).

Liver fibrosis was induced in C57BL/6J mice by intraperitoneal injection CCl_4_ (10% in olive oil) for 6 weeks. As showed in **Figure 1A**, liver tissues from CCl_4_-treated mice presented with fibrotic lesions and obvious hepatomegaly. Hematoxylineeosin (H&E) staining, masson trichrome staining showed that normal lobular architecture with central veins and radiating hepatic cords in the vehicle group, while liver fibrosis liver showed prominent hepatic steatosis, necrosis, formation of regenerative nodules and fibrotic septa (**Figure 1B**). The immunostaining results showed that the expression of α-SMA was extensively stained in CCl_4_-treated mice livers tissue section (**Figure 1C**). Moreover, serum ALT and AST levels were obviously elevated in CCl_4_-induced fibrotic mice compared with vehicle group (**Figure 1D**). The proteins and mRNAs levels of COL1a1 and α-SMA were dramatically elevated while PTGIS was notably decreased in primary HSCs isolated from fibrotic livers relative to the vehicle group (**Figure 1E**). As showed in **Figure 1F**, immunohistochemistry staining pointed out that PTGIS expression was dramatically decreased in fibrotic liver tissues. As showed in Supplementary Figure 1, immunofluorescence staining illustrated that α-SMA protein expression was elevated in primary HSCs isolated from CCl_4_-treated mice group compared with vehicle-treated group. Double immunofluorescence staining showed that PTGIS-positive area and α-SMA-positive area were highly overlapped (**Figure 1G**), which indicated that PTGIS might be expressed in HSCs. Taken together, these data indicated that liver fibrosis mice model were well established and PTGIS expression was decreased in CCl_4_-induced mice model.

### PTGIS Expression Was Increased in the First 4 weeks and Then Decreased in the Process of Model Establishment

**FIGURE 2:**
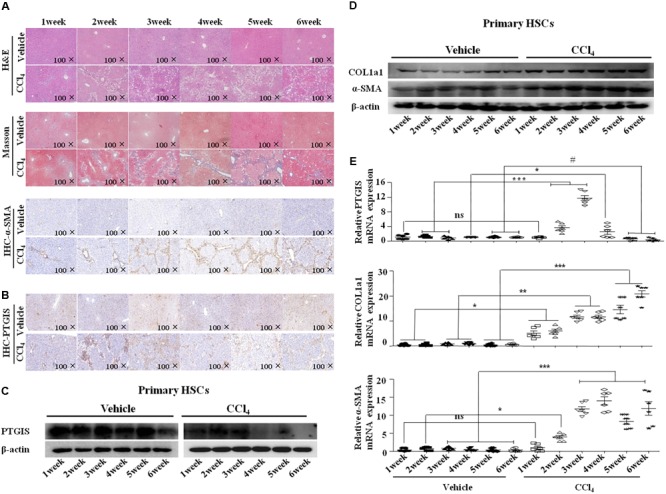
PTGIS expression changes between the model establishment. **(A)** The mice were stimulated with CCl_4_ for different time span. Pathology observation of the mouse liver sections stained with H&E staining, Masson staining (100×). The expression of α-SMA were detected by immunohistochemistry(100×). **(B)** Immune signals for PTGIS (100×). **(C)** The protein expression changes of PTGIS in vehicle and CCl4-treated group was measured by Western blot experiment. **(D)** The protein levels of PTGIS, COL1a1 and α-SMA were determined by western blot analysis. **(E)** PTGIS, COL1a1 and α-SMA mRNA expression levels were detected by RT-qPCR, ^∗^*P* < 0.05, ^∗∗^*P* < 0.01, ^∗∗∗^*P* < 0.001 vs. vehicle group.

To detected the PTGIS expression changes in the process of fibrotic models establishment, C57BL/6J mice were stimulated with CCl_4_ (10%) with different time points (1, 2, 3, 4, 5, 6 week). Hematoxylineeosin (H&E) staining, masson trichrome staining, immunohistochemistry (IHC) for α-SMA showed that the damage of tissue were enhanced by time-dependent manner (**Figure 2A**). Interestingly, immunohistochemistry (IHC) for PTGIS revealed that PTGIS were obviously increased in early-stage CCl_4_-induced liver injury, and remarkably decreased after 4 weeks CCl_4_ induced fibrotic livers compared with 3 weeks (**Figure 2B**). Additionally, Western Blot results revealed that COL1a1 and α-SMA protein levels were dramatically increased in a time-depend manner (**Figure 2D**) and the protein levels of PTGIS was increased firstly and decreased after 4 weeks of CCl_4_-treated (**Figure 2C**). Furthermore, the mRNA levels of PTGIS, COL1a1 and α-SMA were consistent with Western blot analysis (**Figure 2E**). It has been demonstrated that PTGIS own anti-inflammatory property (Phillips et al., 2014; Cebola et al., 2015). Therefore, we supposed that the up-regulated expression of PTGIS in early-fibrotic-stage was a positive feedback to liver inflammation.

### PTGIS Expression Was Down-Regulated in HSCs *in Vitro*

**FIGURE 3:**
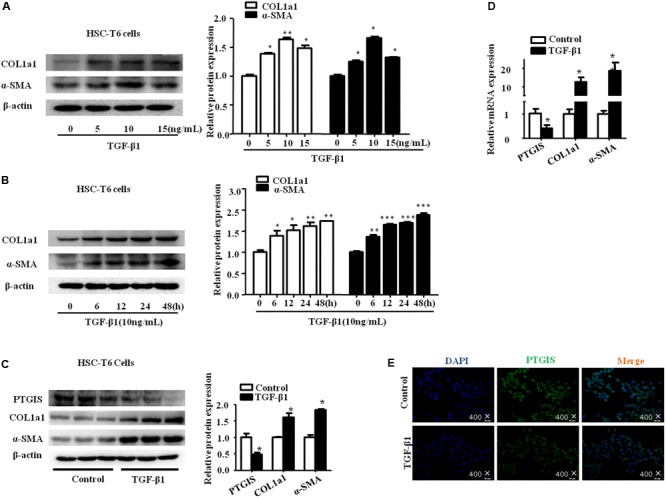
The expression of PTGIS in HSC-T6 cells treated with TGF-β1. **(A)** HSC-T6 cells were stimulated with different concentration of TGF-β1 (0, 5, 10, 15 ng/mL) for 24 h. The protein expression levels of COL1a1 and α-SMA were measured by western blot. The results are shown as relative expression against non-treated HSC-T6 cells. ^∗^*p* < 0.05, ^∗∗^*p* < 0.01 vs. non-treated group. **(B)** HSC-T6 cells were stimulated with TGF-β1 (10 ng/mL) for 0, 6, 12, 24, 48 h. The protein levels of COL1a1 and α-SMA were measured by Western blot, ^∗^*p* < 0.05, ^∗∗^*p* < 0.01, ^∗∗∗^*p* < 0.001 vs. control group. **(C)** The PTGIS, COL1a1 and α-SMA protein levels treated with TGF-β1 (10 ng/mL) for 24 h in HSC-T6 cells were detected by western blot analysis, ^∗^*P* < 0.05, ^∗∗^*P* < 0.01, ^∗∗∗^*P* < 0.001 vs. control group. **(D)** The mRNA levels of PTGIS, COL1a1 and α-SMA were measured by RT-qPCR experiment, ^∗^*P* < 0.05 vs. control group. **(E)** The expression levels of PTGIS protein in the HSC-T6 cells treated with TGF-β1 were determined by Immunofluorescence (400×).

To determine the expression of PTGIS in TGF-β1-treated HSC-T6 cells, we performed Western blot, RT-qPCR and immunoflurescence staining. As illustrated in **Figure 3A**, HSC-T6 cells treated with TGF-β1 at the concentration of 0, 5 10, 15 ng/mL for 24 h, the results showed that highest levels of COL1a1 and α-SMA was presented at the concentration of 10 ng/mL. Additionally, the expression levels of COL1a1 and α-SMA protein were also up-regulated in HSC-T6 cells stimulated with TGF-β1 (10 ng/mL) for 0, 6, 12, 24, 48 h (**Figure 3B**). Take all the results into consideration, we chose TGF-β1 (10 ng/mL) and 24 h for the further study. As showed in **Figure 3C**, the protein levels of PTGIS were down-regulated in HSC-T6 cells treated with TGF-β1 (10 ng/mL) compared with Control group. The result of RT-qPCR was similar (**Figure 3D**). We further examinated the PTGIS protein expression by immunofluorescence staining (**Figure 3E**), the results showed that PTGIS was expressed in the cytoplasm and was dramatically decreased in TGF-β1-treated group.

### The Down-Regulated PTGIS Expression Was Associated With DNA Hypermethylation and Can Be Restored by 5-azadC and DNMTs-RNAi

**FIGURE 4:**
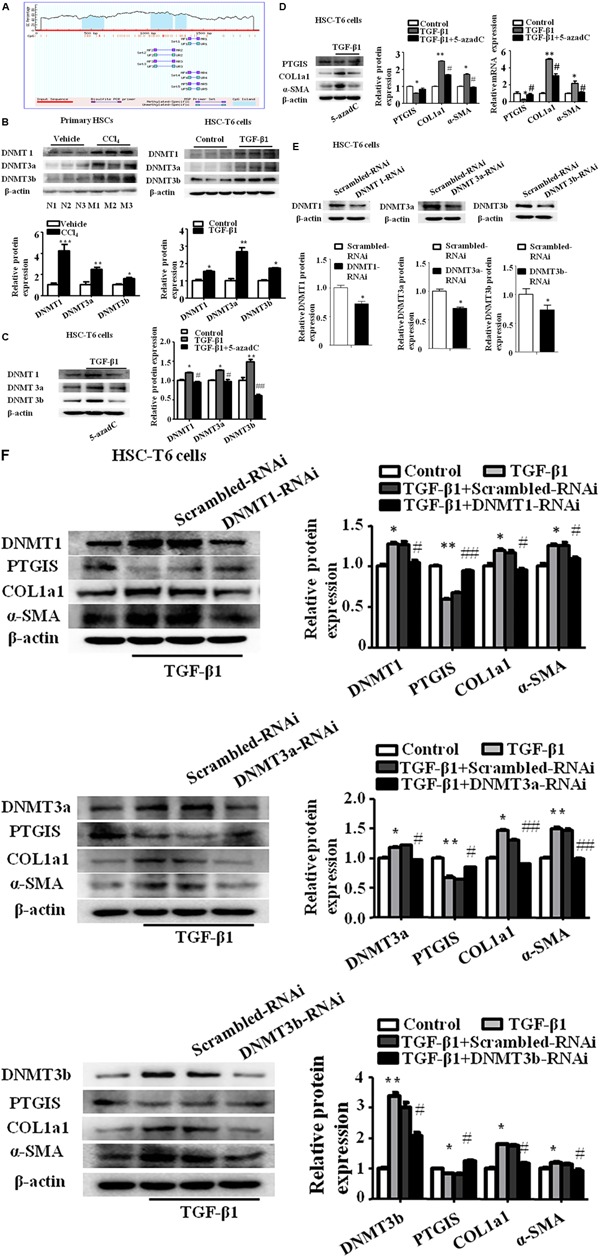
The expression of PTGIS was associated with DNA hypermethylation in activated HSC-T6 cells. **(A)** The CpG island in *PTGIS* gene were predicted. **(B)** The protein levels of DNMT1, DNMT3a, and DNMT3b in primary HSCs isolated from fibrotic livers and HSC-T6 cells were detected by western blot analysis, ^∗^*P* < 0.05, ^∗∗^*P* < 0.01, ^∗∗∗^*P* < 0.001 vs. vehicle group or control group. **(C)** The protein levels of DNMT1, DNMT3a and DNMT3b in control, model and TGF-β1 + 5-azadC groups, HSC-T6 cells were treated with 5-azadC (2 μM) 6 h before added TGF-β1(10 ng/mL) for 24 h, ^∗^*p* < 0.05, ^∗∗^*p* < 0.01 vs. control group, ^#^*p* < 0.05, ^##^*p* < 0.01 vs. TGF-β1-treated group. **(D)** The protein and mRNA levels of PTGIS, COL1a1, and α-SMA were measured by western blot and RT-qPCR, ^∗^*P* < 0.05, ^∗∗^*P* < 0.01 vs. control group, ^#^*p* < 0.05 vs. TGF-β1 group. **(E)** The efficiency of DNMT1, DNMT3a and DNMT3b lose-expression in HSC-T6 cells were detected by western blot, ^∗^*p* < 0.05 vs. Scrambled-RNAi group. **(F)** The protein levels of DNMT1, DNMT3a, DNMT3b, PTGIS, COL1a1 and α-SMA were detected via western blot analysis, ^∗^*P* < 0.1, ^∗∗^*P* < 0.05 vs. control group, ^#^*P* < 0.1, ^##^*P* < 0.05 vs. TGF-β1 + Scrambled-RNAi group.

To determine whether decreased PTGIS expression was associated with DNA methylation in fibrotic livers, prediction was done to determine the CpG island of PTGIS gene (**Figure 4A**). We detected the protein expression of DNMT1, DNMT3a and DNMT3b were detected *in vivo* and *in vitro*. As showed in **Figure 4B**, DNMT1, DNMT3a, and DNMT3b expression were up-regulated in primary HSCs isolated from CCl_4_-induced fibrotic mice and TGF-β1-treated HSC-T6 cells.

To further elucidate whether the lost expression of PTGIS was results from aberrant DNA methylation, the DNMTs inhibitors 5-aza-2′-deoxycytidine (5-azadC) (2 μM) was used 6 h before TGF-β1-treated in HSC-T6 cells (Cai et al., 2016). Western blot results revealed that the protein levels of DNMT1, DNMT3a and DNMT3b were obviously inhibited by 5-azadC (2 μM) (**Figure 4C**). The lost expression of PTGIS was restored by 5-azadC (2 μM) (**Figure 4D**). These data suggested that DNMTs of HSCs may have an important role in the methylation of PTGIS.

To further identify which DNMTs mainly influenced PTGIS promoter methylation, DNMT1-siRNA, DNMT3a-siRNA and DNMT3b-siRNA were used to knockdown DNMT1, DNMT3a and DNMT3b expression in HSC-T6 cells, respectively. Firstly, the inhibitory effect was verified by Western Blot (**Figure 4E**). The results of Western Blot revealed that PTGIS expression were negatively with the protein levels of DNMT1, DNMT3a and DNMT3b, and the protein levels of COL1a1 and α-SMA were negatively related with PTGIS expression (**Figure 4F**). ChIP assay showed that PTGIS gene can be pulled down by anti-DNMT1 and anti-DNMT3b antibody in TGF-β1 activated HSC-T6 cells whereas anti-DNMT3a and negative control anti-IgG antibody cannot pull down PTGIS gene. The results of ChIP assay indicated that DNMT1 and DNMT3b directly binding with PTGIS gene in HSC-T6 cells (Supplementary Figure 2A). MSP analysis showed that PTGIS gene was mainly amplified by PTGIS unmethylated primer (PTGIS-U) in non-treated cells, whereas mainly amplified by PTGIS methylated primer (PTGIS-M) in TGF-β1-treated HSC-T6 cells (Supplementary Figure 2B). The results of MSP showed that TGF-β1 could induced aberrant hypermethylation of PTGIS gene. Taken together, these data indicated that the downregulation of PTGIS in liver fibrosis was attributed to DNA methylation and PTGIS gene methylation was mainly caused by DNMT1 and DNMT3b.

### Liver-Specific PTGIS Overexpression Alleviated CCl_4_-Induced Liver Fibrosis in C57BL/6J Mice

**FIGURE 5:**
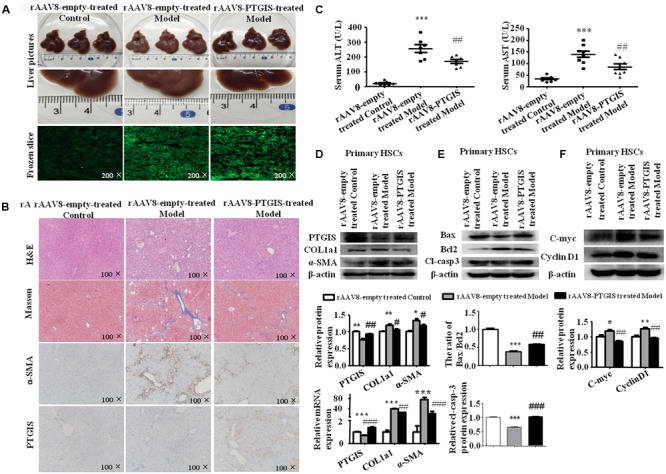
Liver-specific PTGIS overexpression alleviated CCl_4_-induced liver fibrosis. **(A)** Macroscopic examination of fresh liver tissue without fixation from C57BL/6J mice in rAAV8-empty-treated control group, rAAV8-empty-treated model group and rAAV8-PTGIS-treated model group, and the transduction efficient of rAAV8-PTGIS-eGFP in liver tissues were examinated by laser confocal microscopy. **(B)** Pathology observation of the mouse liver sections stained with H&E staining, masson staining (200 μM), immunohistochemistry (IHC) for α-SMA (200 μM) and PTGIS (200 μM). **(C)** Serum ALT and AST levels, ^∗∗∗^*P* < 0.001 vs. rAAV8-empty treated Control group, ^##^*p* < 0.01 vs. rAAV8-empty treated Model group. **(D)** The protein and mRNA expression levels of PTGIS, COL1a1, α-SMA were examinated by western blot and RT-qPCR analysis, ^∗^*P* < 0.05, ^∗∗^*P* < 0.01 vs. rAAV8-empty treated Control group, ^#^*p* < 0.05, ^##^*p* < 0.01 vs. rAAV8-empty treated Model group. **(E)** the protein levels of Bax, Bcl_2_, and Cleaved-caspase3 were assessed by western blot experiment, ^∗∗∗^*P* < 0.001 vs. rAAV8-empty treated Control group, ^##^*p* < 0.01, ^###^*p* < 0.001 vs. rAAV8-empty treated Model group. **(F)** The protein levels of C-myc and CyclinD1 were detected by western blot analysis, ^∗^*P* < 0.05 vs. rAAV8-empty treated Control group, ^#^*p* < 0.05 vs. rAAV8-empty treated Model group.

Recombinant adeno-associated viral (rAAV) vectors can infect both dividing and non-dividing cells *in vitro* and *in vivo*, establishing long-term and efficient transgene expression with minimal toxicity and cellular immune response (Gao et al., 2002; McCarty et al., 2003; Wang et al., 2003; McCarty, 2008). To identify the function of PTGIS in liver fibrosis *in vivo*, rAAV8-empty or rAAV8-PTGIS was intravenously injected into CCl_4_-treated mice via the tail vein before the first injection of 10% CCl_4_. Fluorescent microscopy showed efficient transduction of liver, as indicated by eGFP expression (**Figure 5A**). Histologically, H&E staining and Masson trichrome staining showed that forced expression of PTGIS could alleviate steatosis, necrosis and collagen deposition (**Figure 5B**). IHC-PTGIS showed that the PTGIS expression was restored in primary HSCs after administration of rAAV8-PTGIS in model group (**Figure 5B**). Furthermore, forced expression of PTGIS dramatically decreased the levels of serum ALT/AST compared with model group (**Figure 5C**). The protein and mRNA expression levels of COL1a1 and α-SMA were down-regulated in primary HSCs isolated from rAAV8-PTGIS-treated model group compared with rAAV8-empty-treated group (**Figure 5D**). The data presented indicated that adding PTGIS expression *in vivo* could alleviate CCl_4_-induced HSCs activation and liver fibrosis in mouse model.

### PTGIS Overexpression Inhibited TGF-β1-Induced HSC-T6 Cells Activation and Promoted Activated HSCs Apoptosis *in Vivo* and *in Vitro*

It has been demonstrated previously that PTGIS can inhibit cell growth in lung to alleviate pulmonary hypertension (Tuder et al., 1999) and overexpression of PTGIS in human embryonic kidney epithelial 293 (HEK-293) cell line can induce cell death (Hatae et al., 2001). Accordingly, we supposed that forced PTGIS expression *in vivo* and *in vitro* may alleviate liver fibrosis through affect HSCs activation and survival. To test this hypothesis, we examinated the expression levels of apoptosis associated protein (Bax, Bcl_2_, and cleaved-casepase3) and Cell cycle-related protein (C-myc and CyclinD1) in primary HSCs. The Western Blot analysis showed that the ratio of Bax/Bcl_2_ and the expression of cleaved-casepase3 were significantly elevated in primary HSCs isolated from rAAV8-PTGIS-treated model group compared with primary HSCs isolated from rAAV8-empty-treated model group (**Figure 5E**), which indicated that PTGIS may have pro-apoptosis effect on primary HSCs. The increased C-myc and CyclinD1 protein levels in primary HSCs isolated from rAAV8-empty-treated model group were decreased in rAAV8-PTGIS-treated model group (**Figure 5F**). The results indicated that adding PTGIS expression inhibits cell cycle.

**FIGURE 6:**
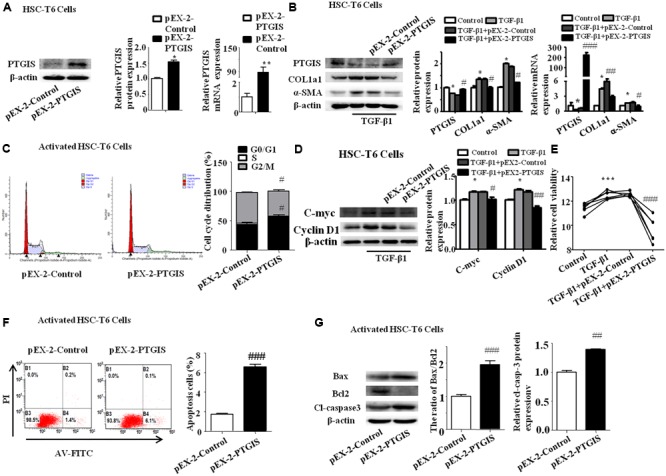
Function of forced PTGIS expression on TGF-β1-induced activation of HSC-T6 cells. **(A)** The efficiency of PTGIS over-expression in HSC-T6 cells were detected by western blot and RT-qPCR analysis. ^∗^*P* < 0.05, ^∗∗^*P* < 0.01 vs. control group. **(B)** The PTGIS, COL1a1 and α-SMA protein and mRNA expression levels in TGF-β1-treated and pEX-2-PTGIS-transfected HSC-T6 cells were detected by western blot and RT-qPCR experiments. ^∗^*P* < 0.05 vs. control group, ^#^*P* < 0.05 vs. TGF-β1 + pEX-2-control group. **(C)** The cell cycle were measured by flow cytometry analysis of pEX-2-PTGIS-transfected HSC-T6 cells, ^#^*p* < 0.1 vs. TGF-β1 + pEX-2-control group. **(D)** The protein and mRNA levels of C-myc and Cyclin D1 were detected by western blot analysis, ^∗^*P* < 0.05 vs. control group, ^#^*P* < 0.05 vs. TGF-β1 + pEX-2-control group. **(E)** The relative cell viability of HSC-T6 cells were measured by CCK8 analysis, ^∗∗^ *P* < 0.001 vs. control group, ^###^*P* < 0.001 vs. TGF-β1 + pEX-2-control group. **(F)** FACS analysis were used to examined apoptosis rate alteration in HSC-T6 cells from pEX-2-control and pEX-2-PTGIS group in TGF-β1 activated HSC-T6 cells, ^###^*P* < 0.001 vs. TGF-β1 + pEX-2-control group. **(G)** The apoptosis-associated proteins (Bax/Bcl_2_, cleaved-casepase3) were detected by western blot analysis, ^##^*P* < 0.01, ^###^*P* < 0.001 vs. pEX-2-control group in activated HSC-T6 cells.

To explore the function of PTGIS in the progress of HSC-T6 cells activation, HSC-T6 cells were transfected with PTGIS plasmid (pEX-2-PTGIS). As showed in **Figure 6A**, pEX-2-PTGIS plasmid could obviously elevate PTGIS protein and mRNA expression. Forced-expression of PTGIS significantly decreased the protein and mRNA levels of COL1a1 and α-SMA in pEX-2-PTGIS group compared with pEX-2-control group in TGF-β1 activated HSC-T6 cells (**Figure 6B**). The results of flow cytometric analysis (FCM) showed that over-expression of PTGIS observably induced G_0_/G_1_ arrest in HSC-T6 cells compared with pEX-2-PTGIS-control group, accompanied with an obvious reduction of cell numbers in the G_2_/M phase (**Figure 6C**). In addition, the expression of C-myc and CyclinD1 protein were up-regulated in TGF-β1-treated group and down-regulated in pEX-2-PTGIS group (**Figure 6D**). CCK8 assay suggested that the viability of HSC-T6 cells was obviously increased in TGF-β1-treated group compared with control group and significantly decreased in pEX-2-PTGIS group compared with pEX2-control group (**Figure 6E**). Next, we investigated the influence of PTGIS over-expression on the apoptosis of activated HSC-T6 cells by Annexin-V/PI staining. Flow cytometric analysis revealed that forced PTGIS expression increased apoptosis cells percentage in activated HSC-T6 cells (**Figure 6F**). Furthermore, forced PTGIS expression significantly increased the ratio of Bax/Bcl_2_ and Cleaved-caspase3 levels (**Figure 6G**). The above results demonstrated that overexpression of PTGIS can inhibit HSC-T6 cells activation and promote activated HSC-T6 cells apoptosis *in vitro*.

### PTGIS Silencing Enhanced HSC-T6 Cells Activation *in Vitro*

**FIGURE 7:**
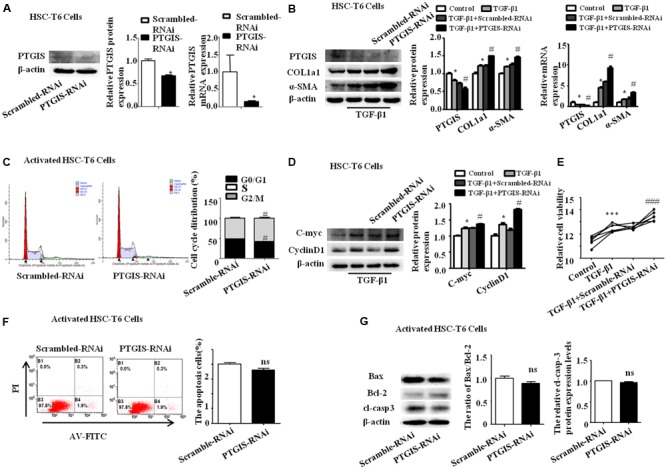
Influence of loss-expression of PTGIS in TGF-β1-treated HSC-T6 cells. **(A)** The efficiency of PTGIS lose-expression in HSC-T6 cells were detected by western blot and RT-qPCR. ^∗^*p* < 0.05 vs. control group. **(B)** The PTGIS, COL1a1 and α-SMA protein and mRNA expression levels in PTGIS-RNAi-transfected HSC-T6 cells were detected by western blot and RT-qPCR experiments. ^∗^*P* < 0.05 vs. control group, ^#^*P* < 0.05 vs. TGF-β1 + Scrambled-RNAi group. **(C)** The cell cycle of PTGIS-RNAi transfected HSC-T6 cells, ^#^*P* < 0.05 vs. TGF-β1 + Scrambled-RNAi transfected group in TGF-β1 activated HSC-T6 cells. **(D)** The protein and mRNA levels of Cmyc and Cyclin D1 were detected by western blot analysis, ^∗^*P* < 0.05 vs. control group, ^#^*p* < 0.05 vs. Scrambled-RNAi transfected group in activated HSC-T6 cells. **(E)** The relative cell viability of HSC-T6 cells were measured by CCK8 analysis, ^∗∗∗^*P* < 0.001 vs. control group, ^###^*p* < 0.001 vs. TGF-β1 + Scrambled-RNAi transfected group. **(F)** FACS analysis were used to examined apoptosis rate alteration in HSC-T6 cells from Scrambled-RNAi transfected group and PTGIS-RNAi transfected group in TGF-β1 activated HSC-T6 cells, ^###^*p* < 0.001 vs. TGF-β1 + Scrambled-RNAi transfected group. **(G)** The apoptosis-associated proteins (Bax, Bcl_2_, cleaved-casepase3) were detected by western blot analysis, ^##^*p* < 0.01, ^###^*p* < 0.001 vs. Scrambled-RNAi transfected group in activated HSC-T6 cells.

PTGIS-RNAi was used further to confirm the effect of PTGIS on the progress of HSC-T6 cells activation. Western Blot and RT-qPCR results indicated that PTGIS-RNAi could successfully silenced the expression of PTGIS (**Figure 7A**). As presented in **Figure 7B**, the expression of COL1a1 and α-SMA were significantly up-regulated in the protein and mRNA levels in the PTGIS-RNAi transfected group compared with scrambled-RNAi group in activated HSC-T6 cells. The results of flow cytometric analysis (FCM) showed that inhibited PTGIS expression could increase the percentage of G_2_/M in activated HSC-T6 cells (**Figure 7C**). As showed in **Figure 7D**, the protein levels of C-myc and CyclinD1 were significantly increased in PTGIS-RNAi group compared with Scrambled-RNAi group in TGF-β1-activated HSC-T6 cells. CCK8 assay suggested that the viability of HSC-T6 cells was significantly elevated PTGIS-RNAi group contrasted with scrambled-RNAi group (**Figure 7E**). Flow cytometric analysis revealed that lose-expression of PTGIS had no significant influcence on the apoptotic cell numbers in activated HSC-T6 cells (**Figure 7F**). Furthermore, losting PTGIS expression had no influence on the ratio of Bax/Bcl-2 and Cleaved-caspase3 expression (**Figure 7G**). The possible reasons for why PTGIS silencing has no effect on apoptosis may be is the expression of PTGIS was too little to influence the apoptosis process of activated HSC-T6 cells. Taken together, these data indicated that PTGIS silencing could enhance activation of HSC-T6 cells but have little influence on HSC-T6 cells activation.

## Discussion

Liver fibrosis is a medical condition characterized by an extensive deposition of extracellular compounds. With extensive accumulation of ECM, liver fibrosis will ultimately developed into liver cirrosis and HCC, which requiring for liver transplant (Bataller and Brenner, 2005; Jiao et al., 2009; Troeger et al., 2012). Therefore, more and more studies paid attention to hepatic fibrosis not only there is no effective therapeutic strategy for liver fibrosis but also it will lead serious consequence. HSCs-derived myofibroblast is the principle source of ECM, which plays pivotal role in the pathogenesis of liver fibrosis (Hernandez-Gea and Friedman, 2011). For a long time, hepatic fibrosis was thought to be an irreparable pathological process because of the collapse of liver parenchyma and vascular architecture (Popper and Uenfriend, 1970). Nevertheless, increasingly clinical and experiment researchers have underlined that established liver fibrosis can be reversed if remove the etiological agents and promote the activated HSCs apoptosis (Ellis and Mann, 2012; Chen et al., 2016). Therefore, inhibiting HSCs activation and promoting activated HSCs apoptosis to blocking ECM deposition, finally remolding the distorted liver architecture will be the crucial therapeutic strategy for liver fibrosis. In our study, force- and gain-experiment of PTGIS *in vivo* and *in vitro* revealed a closely correlation between PTGIS and HSCs activation as measured by the expression of α-SMA and COL1a1.

Epigenetic modification plays a pivotal role in many physiological and pathological process, including tumor genesis and fibrogenesis (Bian et al., 2014, 2017; Wu et al., 2017). The genomic methylation screening results shows that PTGIS promoter was hypermethylated in fibrotic mice. In our study, we demonstrated that DNMTs protein levels were elevated in mice induced with CCl_4_ and HSC-T6 cells treated with TGF-β1. And decreased PTGIS expression can be restores by DNMT-RNAi and 5-azadC. The results of MSP confirmed that PTGIS was hypermethylated in HSC-T6 cells cultured with TGF-β1 (10 ng/mL). The ChIP assay indicated that the methylation of PTGIS was mainly attributed to DNMT1 and DNMT3b. Taken together, these data indicated that downregulation of PTGIS in hepatic fibrosis was attributed to hypermethylation of PTGIS promoter which mainly induced by DNMT1 and DNMT3b.

**FIGURE 8:**
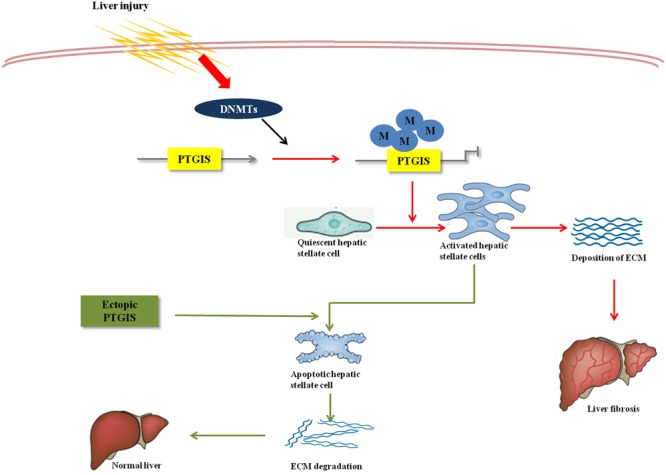
Overview of PTGIS in liver fibrosis. The major mechanism contributed to PTGIS exerts its function in hepatic fibrogenesis. DNA methyltransferases led to predominant inhibition of PTGIS expression. Ectopic expression of PTGIS alleviate liver fibrosis by promoting activated hepatic stellate cells apoptosis and then reverse the progress of liver fibrosis.

Forced PTGIS expression *in vivo* through tail vein injection rAAV8-PTGIS can alleviate liver fibrosis, which was certified by the decreased COL1a1 and α-SMA protein and mRNA expression levels in primary HSCs isolated from fibrotic mice and the downregulated serum ALT/AST levels. Moreover, the functional studies revealed that forced-expression of PTGIS mediated by pEX2-PTGIS plasmid *in vitro* can inhibit HSC-T6 cells activation and promote activated HSC-T6 cells apoptosis. However, loss-expression of PTGIS can promote HSC-T6 cells activation and ECM accumulation. The elevated apoptosis-related protein in forced PTGIS expression group *in vivo* and *in vitro* verified that PTGIS induced activated HSCs apoptosis. This verdict was further attested by FCM results *in vitro* in pEX2-PTGIS transfected group. The major mechanism contributed to PTGIS exerts its function in hepatic fibrogenesis was showed in **Figure 8**.

It is interesting to note that PTGIS was elevated in the early-liver-fibrotic stage and down-regulated in the later period of model establishment process in the process of fibrotic mice model establishment. It has been demonstrated that PTGIS was hypermethylated in colorectal cancer and misregulation of PTGIS leads to the accumulation of pro-inflammatory signals (Cebola et al., 2015). Furthermore, most type of liver insult damage epithelial cells, which leads to the release of inflammatory cytokines such as TGF-β1, TNF, IL-6 and IL-1β (Pellicoro et al., 2014). Then HSCs activated by TGF-β1. Therefore, we hypothesized that the elevated PTGIS expression in the early-liver-fibrotic stage may attribute to its positive feedback to inflammation response.

## Conclusion

Our experiment results suggested that DNA methylation of PTGIS plays a pivotal role in the progression of liver fibrosis and HSCs activation. Forced PTGIS expression *in vivo* and *in vitro* can offset CCl_4_ or TGF-β-induced activation of HSCs, and even provoke cells apoptosis. However, PTGIS silencing facilitate the activation of HSCs and ECM deposition. These results indicated a latent therapeutic capacity of PTGIS for treatment of liver fibrosis. As far as we know, this is the first investigation about PTGIS hypermethylation in progression of liver fibrosis *in vivo* and *in vitro*. This study suggests a therapeutic role of PTGIS in liver fibrosis and encourages further studies to determine the value of PTGIS as a novel biomarker in hepatic fibrosis.

Despite the fact that HSCs play a pivotal role in liver fibrosis, HC is the domain cell type residing in the liver and HCs apoptosis and impaired HCs proliferation also have been commonly recognized as critical inhibitors of fibrosis. Persistence of chronic inflammation response always resulted in hepatic fibrosis (Friedman, 2008b; Sacchi et al., 2015; Zhang et al., 2017), and kupffer cells plays important role in trigger the process of hepatic fibrosis. It has been reported that PGI_2_, the product of PTGIS, promotes colorectal cancer growth probably by activating PPARδ (Gupta et al., 2000) and inhibition of COX-2-derived PGI_2_ induces colon cancer cells apoptosis (Wu and Liou, 2009). Additionally, it also reported that PGI_2_ regulates the transcription of VEGF by PPARδ (Mizukami et al., 2004; Arany et al., 2008) and VEGF expression is augment by hypoxia-induced PTGIS in human fibroblast (Wang et al., 2013). We detected the expression of PTGIS protein in primary hepatocytes and primary macrophages isolated from mice treated with CCl4 (Supplementary Figures 3A,B). The results of Western blot showed that PTGIS protein expression was significantly down-regulated in primary macrophages. In general, it is worth doing further studies to explore whether PTGIS have effect on HCs apoptosis and kupffer cells activation and the underlying molecular mechanism in the progress of liver fibrosis.

## Author Contributions

X-yP performed the experiments, analyzed the data, and drafted the paper. YY, H-dL and XC helped to design the study and helped to draft the paper. H-mH helped to analyze the data. H-wM helped to perform western blot analysis. F-tB, H-xY, and QW helped to isolate primary HSCs. CH and X-mM participated in the design of the study. JL conceived the study and revised the manuscript.

## Conflict of Interest Statement

The authors declare that the research was conducted in the absence of any commercial or financial relationships that could be construed as a potential conflict of interest.

## Abbreviations

5-azadC: 5-aza-2-deoxycytidine
ALT: alanine aminotransferase
AST: aspartate aminotransferase
BCA: bicinchoninic acid
BSA: bovine serum albumin
ChIP: Chromatin immunoprecipitation
DMEM: Dulbecco’s Modified Eagle’s Medium
DNMTs: DNA methyltransferase
ECM: extracellular matrix
FBS: Fetal bovine serum
H&E: hematoxylin and eosin
HC: hepatocyte
HCC: hepatic carcinoma
HIF: human intestinal fibroblasts
HSCs: hepatic stellate cells
IL-1β: interleukin-1β
IL-6: interleukin-6
MSP: methylation-specific PCR
PGH_2_: prostaglandin H_2_
PGI_2_: prostaglandin I_2_
PPARδ: peroxisome proliferator-activator δ
PTGIS: prostacyclin synthase
PVDF: polyvinylidene fluoride
rAAV: Recombinant adeno-associated viral vectors
RT-qPCR: reverse transcription polymerase chain reaction
SDS-PAGE: sodium salt-polyacrylamide gel electrophoresis
TBST: tris buffered saline with tween-20
TGF-ββ1: Transforming growth factor-β1
TNF: tumor necrosis factor
VEGF: vascular endothelial growth factor
