# Supplementary figures and images for: DNA Methylation of PTGIS Enhances Hepatic Stellate Cells Activation and Liver Fibrogenesis
Source: Front Pharmacol. 2018 May 28;9:553. doi: 10.3389/fphar.2018.00553 (PMC5985735; doi:10.3389/fphar.2018.00553)

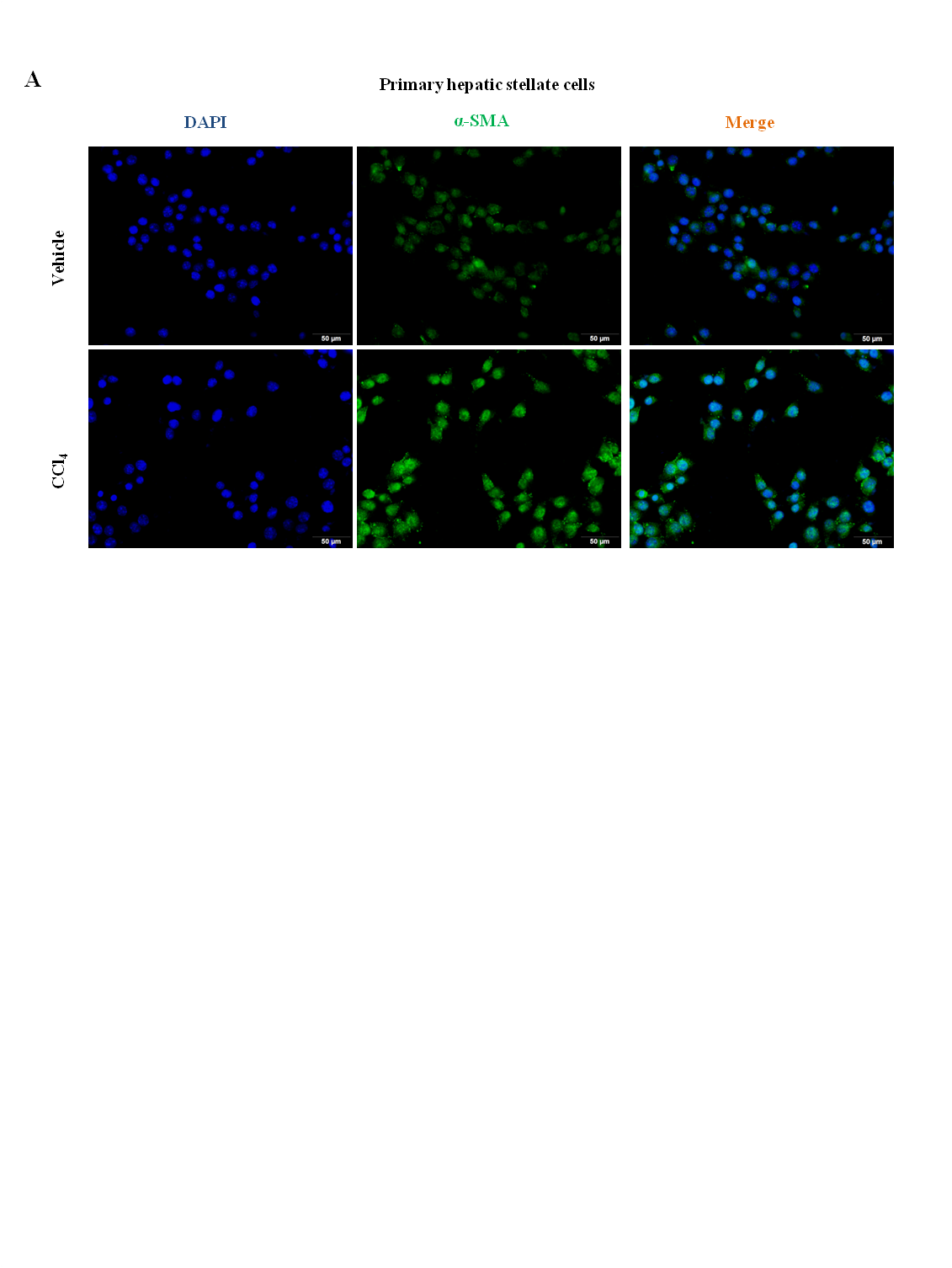

Supplement: Supplementary file 1 [file Image_1.TIF]

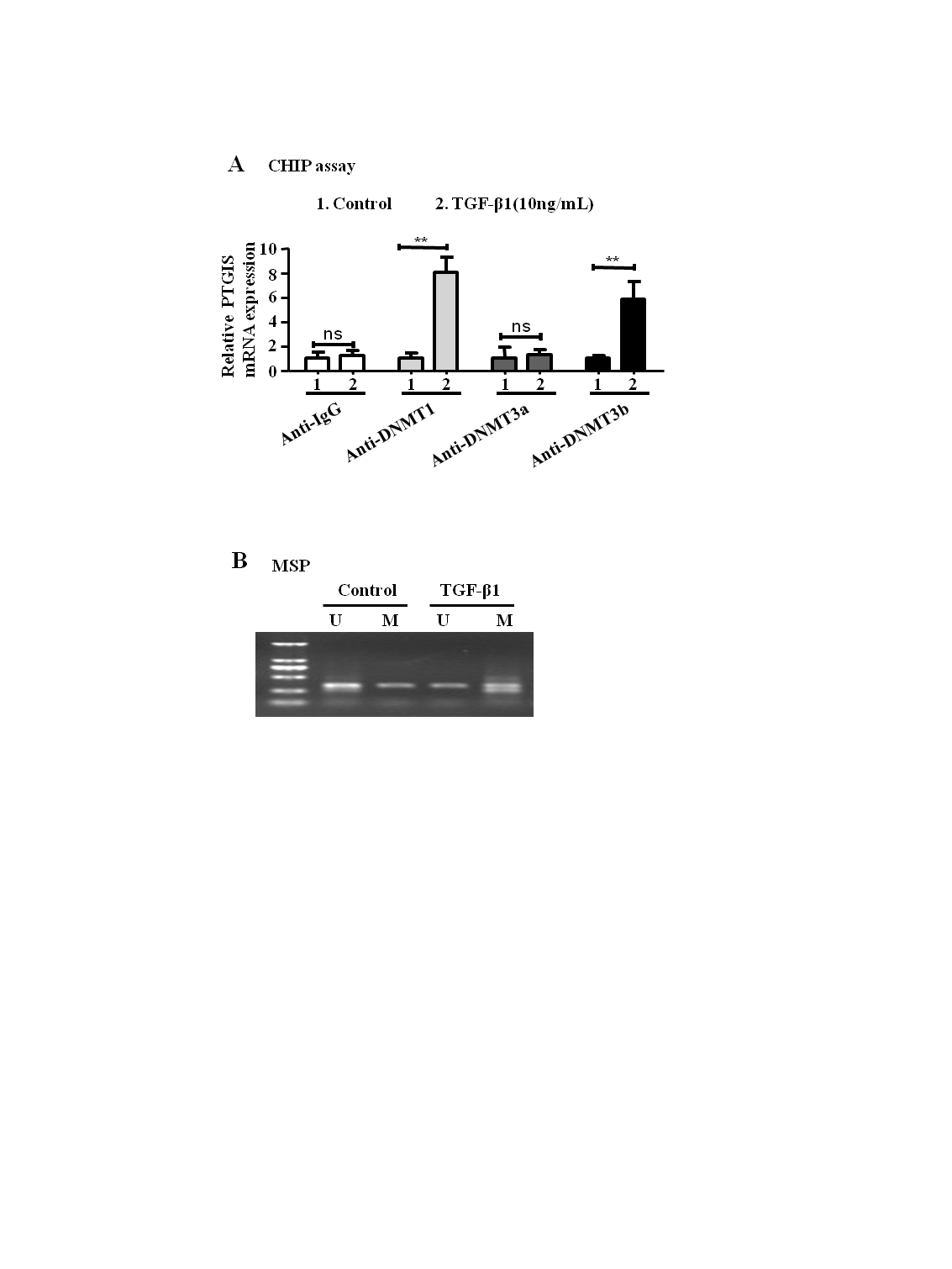

Supplement: Supplementary file 2 [file Image_2.TIF]

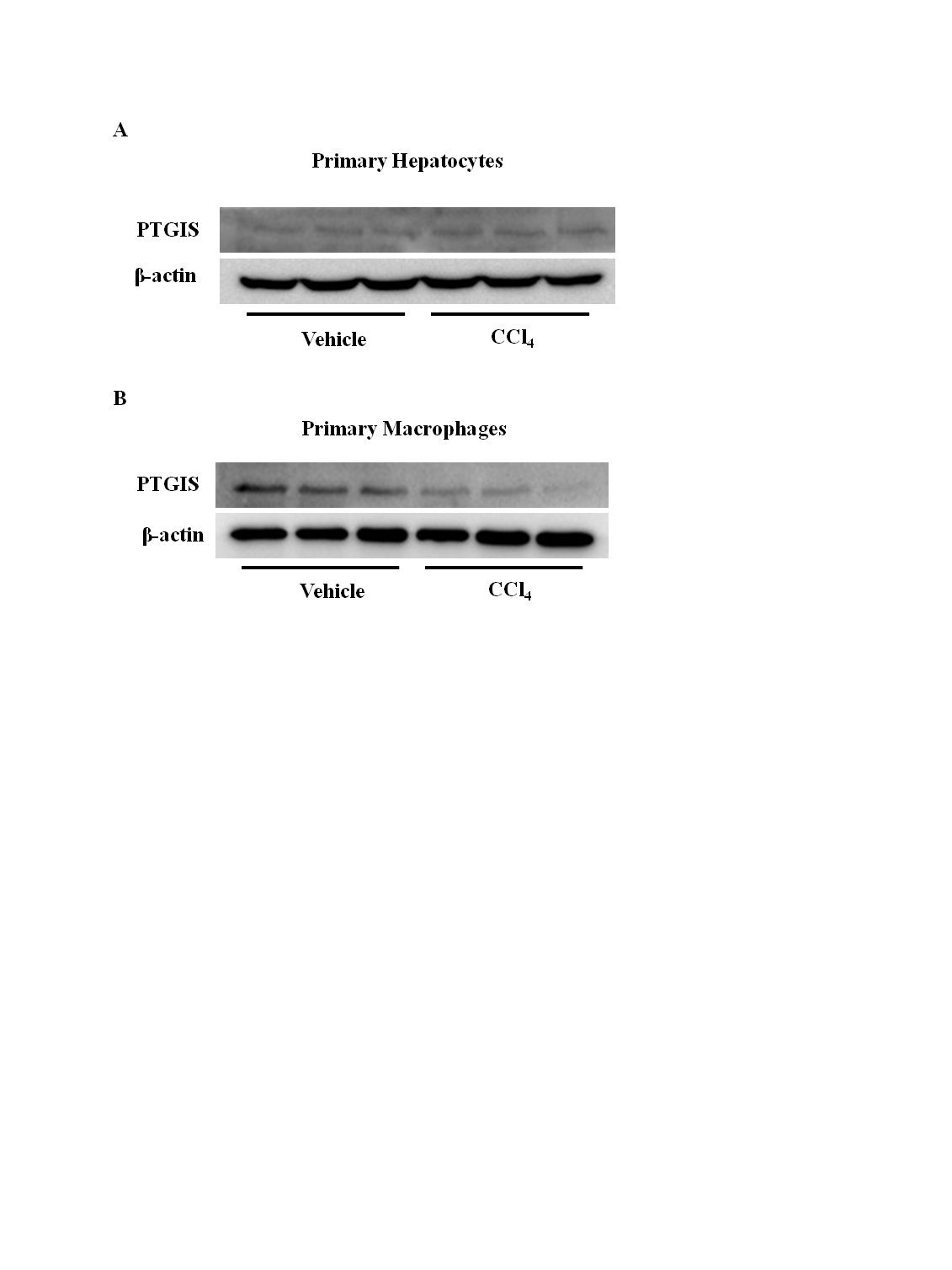

Supplement: Supplementary file 3 [file Image_3.TIF]
