# Supplementary material for: DNA Methylation of PTGIS Enhances Hepatic Stellate Cells Activation and Liver Fibrogenesis
Source: Front Pharmacol. 2018 May 28;9:553. doi: 10.3389/fphar.2018.00553 (PMC5985735; doi:10.3389/fphar.2018.00553)
Supplement: Supplementary file 4 [file Image_4.PDF]

Supplementary Figure Legends:

Supplementary Figure 1. (**A**) The α-SMA protein expression in primary stellated cells isolated from Vehicle and CCl_4-treated mice were determined by Immunofluorescence (400×). Representative views from each group are presented.

Supplementary Figure 2. (**A**) ChIP assay showing the interaction of DNMT1, DNMT3a and DNMT3b with the potential binding sites in the PTGIS promoter. Real-time PCR was performed to detect the amounts of immunoprecipitated product. ns. indicated nonsignificance, $^{**}p<0.01$ vs. Control group. (**B**) MSP analysis of PTGIS in HSC-T6 cells challenged with or without TGF-β1(10ng/mL). All the two groups showed the presence of both unmethylated and methylated DNA respectively. The bands detected by the methylated primer represent methylated PTGIS (M), and the bands detected by the unmethylated primer represent unmethylated PTGIS (U).

Supplementary Figure 3. The PTGIS protein expression in primary hepatocytes (**A**) and primary macrophages (**B**) were measured by Western blot analysis.
