# Supplementary material for: DNA Methylation of PTGIS Enhances Hepatic Stellate Cells Activation and Liver Fibrogenesis
Source: Front Pharmacol. 2018 May 28;9:553. doi: 10.3389/fphar.2018.00553 (PMC5985735; doi:10.3389/fphar.2018.00553)
Supplement: Supplementary file 5 [file Image_5.PDF]

Supplementary Materials and Methods

Methylation-specific PCR

Methylation-specific PCR (MSP) was used to determine the methylation status of 5'regulatory region of PTGIS in non-treated and TGF-β1(10ng/mL)-treated HSC-T6 cells using bisulphite-modified genomic DNA. In brief, 1 ug DNA was denatured by NaOH and modified by sodium bisulphite. The DNA sample was then purified with Wizard DNA purification resin (Promega), treated again with NaOH, ethanol precipitated, and resuspended in H_2O. Methylated and unmethylated DNA specific primer sets were used to investigate the methylated status of 5'regulatory region of PTGIS. The primers used for MSP: PTGIS-U, forward: 5'-GGGATTGAATTTAGGGTTTTGT-3', reverse, 5'-TTATCTAAAAACAACCAACAACATA-3'; PTGIS-M, forward: 5'-ATCGAATTTAGGGTTTTGCGT-3', reverse, 5'-TATCTAAAAACAACCGACGACGTA-3'.

Chromatin immunoprecipitation assay

HSC-T6 cells cultured with TGF-β1(10ng/mL) and relative control cells were cultured at ~80-90% confluency. Subsequently, chromatin immunoprecipitation (ChIP) assay was performed using the SimpleChIP® Enzymatic Chromatin IP Kit (Magnetic Beads) #9003 (Cell Signaling Technology, USA) according to the Manufacturer instruction. The following antibodies were utilized to immunoprecipitate crosslinked protein-DNA complexes: rabbit anti-DNMT1, rabbit anti-DNMT3a, rabbit

anti-DNMT3b and normal rabbit IgG. The immunoprecipitated DNA was purified for PCR analyses with promers specific for the putative binding sites within the promoter of PTGIS.
